# COVID-19 testing and mental health service utilization in Ontario: a population-based cohort study

**DOI:** 10.3389/ijph.2026.1609134

**Published:** 2026-06-02

**Authors:** Kiran Saqib, Vivek Goel, Joel A. Dubin, Jeremy VanderDoes, Zahid Ahmad Butt

**Affiliations:** 1 School of Public Health Sciences, University of Waterloo, Waterloo, ON, Canada; 2 School of Pharmacy, University of Waterloo, Waterloo, ON, Canada; 3 Department of Statistics and Actuarial Science, University of Waterloo, Waterloo, ON, Canada

**Keywords:** anxiety, COVID-19, depression, health administrative data, mental health

## Abstract

**Objective:**

To examine differences in mental health–related healthcare utilization for anxiety and depression between individuals who did and did not undergo COVID-19 PCR testing in Ontario. Background: The COVID-19 pandemic has been associated with changes in mental health and healthcare utilization.

**Methods:**

We conducted a population-based retrospective cohort study using linked ICES data, including 6,175,114 adults (January 2020–March 2021). Exposure was PCR-positive, PCR-negative, or untested. The outcome was time to first mental health–related healthcare use for anxiety and depression, identified using validated codes. Adjusted hazard ratios (aHRs) were estimated using Cox models with propensity score matching.

**Results:**

Individuals who underwent testing had higher mental health–related healthcare utilization than untested individuals. This was observed in PCR-positive (aHR 6.37; 95% CI 6.25–6.50) and PCR-negative groups (aHR 5.91; 95% CI 5.87–5.95). Higher utilization occurred among younger individuals, females, and socioeconomically disadvantaged groups. Results were consistent in matched analyses.

**Conclusion:**

Individuals underwent testing had higher mental health service utilization; similar estimates across PCR-positive and PCR-negative groups suggest testing reflects underlying vulnerability and healthcare-seeking behavior rather than a causal effect on mental health outcomes.

## Introduction

The COVID-19 pandemic and the restrictive measures implemented led to a surge in mental health issues globally [1], including anxiety and depression [2–4]. According to a World Health Organization (WHO) research brief, anxiety and depression increased by 25% in the first year of the pandemic [5]. A global study reported almost 76 million cases of anxiety and 53 million cases of major depressive disorder due to the pandemic, resulting in a 26% and 28% increase, respectively [6]. Mental health has long been recognized to be impacted by epidemics and pandemics; the mental issues caused by viral outbreaks have been dubbed a “parallel epidemic” [7]. The rise in the incidence and prevalence of anxiety and depression was reported as an outcome of the COVID-19 pandemic, and mental health professionals issued a warning of an “echo pandemic” [8] of mental health issues [9]. Concerns surrounding mental health during COVID-19 could be viewed as an epidemic instead of just a delayed outcome of the pandemic [10].

Economic and social mitigation strategies implemented in Canada and other developed countries during the COVID-19 pandemic had unintended consequences that disproportionately affected vulnerable populations. These measures exacerbated existing socioeconomic inequalities, increasing exposure to poverty and its associated mental health impacts, including anxiety and depression [11, 12]. Evidence indicates that low-income individuals, racial and ethnic minorities, and those with limited access to healthcare experienced a disproportionate burden of COVID-19 infection and adverse outcomes [13, 14]. These groups were also more likely to face economic instability, food insecurity, housing challenges, and barriers to mental health services [15]. The intersecting factors including socioeconomic status, pre-existing health conditions, access to care, living conditions, sex, and age, contribute to heightened vulnerability and widening disparities in mental health outcomes [11–13].

Testing for COVID-19 has played a crucial role in identifying and managing the spread of the virus, encompassing various dimensions that highlight its role as an exposure variable [14, 15]. Primarily, it serves as a proxy for direct exposure to the disease, with individuals seeking testing typically driven either by symptoms or close contact with confirmed cases, thus facing the risk of contracting COVID-19 disease. This inherent risk can induce anxiety and stress, as individuals struggle with the uncertainty surrounding their health status and the potential ramifications of a positive test result. The fear of a positive result and its implications on one’s health, livelihood, and relationships can significantly impact mental wellbeing. It is crucial to recognize and address the associated psychological distress that testing can induce. Anxiety, fear of the unknown, stigma, discomfort, information overload, and post-traumatic stress symptoms are among the psychological distress factors associated with COVID-19 testing [12, 16–18]. Overall, while testing may not capture all aspects of disease exposure, it serves as a practical and informative proxy variable that can provide valuable insights into the dynamics of disease transmission and its impact on populations.

Administrative health databases provide comprehensive, population-level data that enable the examination of healthcare utilization patterns during the COVID-19 pandemic. Beyond infection itself, the pandemic introduced multiple stressors including uncertainty, perceived risk of illness, social disruption, and changes in healthcare access that may influence mental health and healthcare-seeking behavior. COVID-19 testing represents a key point of interaction with the healthcare system and may therefore serve as a proxy for underlying psychological vulnerability, perceived risk, and engagement with care. Integrating COVID-19 testing data with other health information allows for a more comprehensive assessment of how these factors relate to mental health service utilization.

Therefore, the objective of this study is to examine the differences in mental health–related healthcare utilization for anxiety and depression between individuals who did and did not undergo COVID-19 PCR testing in Ontario, Canada.

## Methods

### Data source

This study utilized secondary data from administrative health databases accessed through the Ontario Health Database Platform (OHDP). The platform permitted access to patient-level linked population-based health administrative data held at the Institute for Clinical Evaluative Sciences (ICES). The ICES data repository consists of record-level, coded, and linkable health data sets that integrate health services data, registries, and surveys. ICES is an independent, non-profit research institute that houses routinely collected health data from Ontario’s publicly funded healthcare system.

All Ontario residents covered by the province’s universal health coverage were identified using the Registered Persons Database (RPDB), which provided age, sex, and demographic information. COVID-19 status was determined using C19INTGR, a comprehensive dataset of PCR COVID-19 diagnostic results in Ontario. Chronic conditions such as asthma, hypertension, diabetes, COPD, and congestive heart failure were identified using ICES-derived cohorts. These datasets were linked using unique encoded identifiers and analyzed at ICES (Supplementary Tables S3, S4). This study followed the Reporting of Studies Conducted Using Strengthening the Reporting of Observational studies in Epidemiology (STROBE) reporting guidelines [19]. The codes for identifying mental health visits are used for mental health system performance measurement in Ontario and elsewhere in Canada [20–24].

### Study design, settings, and cohort eligibility

**FIGURE 1:**
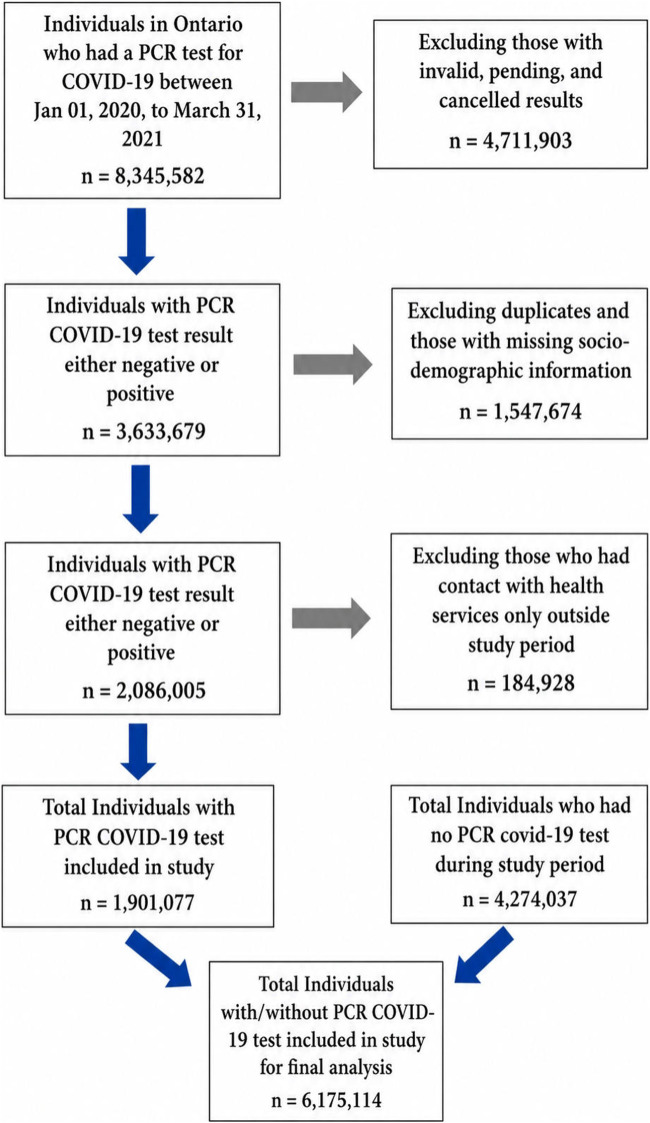
Flow chart of study inclusion and exclusion for population in Ontario (Ontario, Canada, 2021).

We constructed a retrospective cohort of people who were living in Ontario, Canada, had a valid health card, and for whom information on sex and age was available between the period of January 2020 to March 2021 (Figure 1). All those cases who were not Ontario residents or were less than 18 years of age, as well as those who had a standard COVID-19 lab-based PCR test result after the study end period, i.e., beyond 31 March 2021, were excluded. We utilized the National Ambulatory Care Reporting System (NACRS) and the Ontario Health Insurance Plan (OHIP) to identify physician, outpatient visits, and emergency department visits for mental health, and we used the Canadian Institute for Health Information’s Discharge Abstract Database (DAD) and Ontario Mental Health Reporting System (OMHRS) to capture psychiatric hospitalizations. Throughout the study period, COVID-19 testing guidelines were applicable to the general population in Ontario and were not limited to individuals considered at high risk. This helped to maintain a focus on the general population and ensure that our findings accurately reflect the broader impact of the pandemic on Ontario residents.

The exposure in this study was COVID-19 testing by standard lab-based PCR tests in Ontario between January 2020 and March 2021, irrespective of COVID-19 disease status, leading to three groups for comparison: (i) those PCR tested with a positive COVID result; (ii) those PCR tested with a negative COVID result, and (iii) those who were not PCR tested. The event of interest was the time in months until first recorded health services utilization related to anxiety and depression, using DSM-V criteria, ICD-10 codes, and OHIP diagnostic codes during the study period. Individuals who were not PCR tested were selected as the reference group to provide a population-level comparator reflecting mental health–related healthcare utilization among those who did not engage with COVID-19 testing services during the study period. This approach allows for the evaluation of differences associated with testing exposure within the broader population context. However, we acknowledge that untested individuals may differ systematically from those who were tested, particularly in terms of healthcare-seeking behavior, access to care, and underlying health concerns. To mitigate these differences, we applied propensity score matching to balance measured sociodemographic and clinical characteristics between groups. The participants were censored if there was no utilization of anxiety/depression-related health services by the end of the study follow-up period on 31 March 2021. Also, those who died during the study period were censored at the date of death, assuming the event of interest was not experienced first.

### Variables

#### Clinical variables

COVID-19 status was based on a standard lab-based PCR test, while the prevalence of chronic conditions was identified using ICES-derived cohorts. Details of each chronic condition and algorithm used are described in the Supplementary Material (Supplementary Tables S3, S5).

#### Service utilization variables

We utilized an algorithm [25, 26] to identify cases of health service utilization among the Ontario population aged ≥18 years within the ICES data holdings, defined as: (i) one hospitalization in OMHRS or DAD with a primary discharge diagnosis of either anxiety, depression, and anxiety or depressive episode otherwise not specified (NOS) based on DSM-V criteria and ICD-10 codes, or (ii) at least one physician billing and/or emergency department visits in the OHIP claims database or NACRS with a diagnostic code for anxiety, depression, and anxiety or depressive episode otherwise not specified (NOS) based on DSM-IV criteria and ICD-10 codes. A complete list of the codes used to define the study cohort and the study variables is available in the Supplementary Material (Supplementary Tables S3, S5).

#### Sociodemographic variables

We examined sociodemographic variables potentially associated with health service utilization including age, sex, place of residence, and neighborhood-level income quintile using the Ontario Marginalization Index (ON-Marg) [27]. ON-Marg allows for a comprehensive exploration of multiple dimensions of marginalization in urban and rural areas of Ontario.

### Statistical analysis

Descriptive statistics (e.g., mean, median, range, standard deviation) were calculated for the study sample (N = 6,175,114) (Figure 1). Frequencies for demographic and health service use variables were examined with cross-tabulations across both COVID-19 test-based groups using t-tests and chi-squared statistics (Table 1). Criteria for statistical significance were set at p < 0.05. We followed people with and without COVID-19 standard lab-based PCR tests from January 01, 2020, to March 31, 2021, to estimate survival time. Time was defined as the interval between the COVID-19 PCR test date and an event (i.e., first contact with health services for anxiety and depression during study period).

**TABLE 1:** Demographic characteristics and morbidity information of the study population cohort in Ontario (Ontario, Canada, 2021).

Demographics and morbidity information	Total sample	Individuals without PCR COVID-19 tests	PCR test with COVID negative result	PCR test with COVID positive result
Total n (%)	6175114 (100)	4274037 (69.21)	1755519 (28.43)	145558 (2.36)
Mean age yrs. (±SD)	42.95 (15.49)	43.84 (15.60)	41.07 (15.12)	39.39 (14.47)
Anxiety/depression related health service utilization				
No	5511050 (89.25)	3788384 (61.35)	1587938 (25.72)	134728 (2.18)
Yes	664064 (10.75)	485653 (7.86)	167581 (2.71)	10830 (0.18)
Age groups n (%)				
18–45 years	3569070 (57.80)	2363642 (38.28)	1,108,188 (17.95)	97240 (1.57)
46–65 years	2014498 (32.62)	1454906 (23.56)	518139 (8.39)	41453 (0.67)
66–104 years	591546 (9.58)	455489 (7.38)	129192 (2.09)	6,865 (0.11)
Sex n (%)				
Male	2985377 (48.35)	2136749 (34.60)	778344 (12.60)	70884 (1.14)
Female	3189737 (51.65)	2137288 (34.61)	977175 (15.82)	75274 (1.22)
Rural n (%)				
No	5580516 (90.37)	3845375 (62.27)	1594901 (25.83)	140240 (2.27)
Yes	594598 (9.63)	428662 (6.94)	160618 (2.60)	5,318 (0.09)
Ontario marginalization index (%)				
Dependency quintile				
1	1802531 (29.19)	1230040 (19.92)	519994 (8.42)	52497 (0.85)
2	1281491 (20.75)	882985 (14.30)	365722 (5.92)	32784 (0.53)
3	1078592 (17.47)	749393 (12.14)	305374 (4.95)	23825 (0.39)
4	1011615 (16.38)	708060 (11.47)	283600 (4.59)	19955 (0.32)
5	1000885 (16.21)	703559 (11.39)	280829 (4.55)	16497 (0.27)
Deprivation quintile				
1	1519683 (24.61)	1042701 (16.89)	451348 (7.31)	25634 (0.42)
2	1331132 (21.56)	922903 (14.95)	381542 (6.18)	26687 (0.43)
3	1169663 (18.94)	810983 (13.13)	330316 (5.35)	28364 (0.46)
4	1086791 (17.60)	755009 (12.23)	302288 (4.90)	29494 (0.48)
5	1067845 (17.29)	742441 (12.02)	290025 (4.70)	35379 (0.57)
Instability quintile				
1	1393327 (22.56)	970766 (15.72)	382819 (6.20)	39742 (0.64)
2	1186825 (19.22)	828652 (13.42)	333674 (5.40)	24499 (0.40)
3	1121085 (18.15)	781645 (12.66)	317085 (5.13)	22355 (0.36)
4	1076219 (17.43)	740661 (11.99)	312060 (5.05)	23498 (0.38)
5	1397658 (22.63)	952313 (15.42)	409881 (6.64)	35464 (0.57)
Ethnicity diversity quintile				
1	920772 (14.91)	654464 (10.60)	257398 (4.17)	8,910 (0.14)
2	998212 (16.17)	691866 (11.20)	293106 (4.75)	13240 (0.21)
3	1114619 (18.05)	758660 (12.29)	336714 (5.45)	19245 (0.31)
4	1349162 (21.85)	919897 (14.90)	398032 (6.45)	31233 (0.51)
5	1792349 (29.03)	1249150 (20.23)	470269 (7.62)	72930 (1.18)
Asthma				
No	6118287 (99.08)	4240424 (68.67)	1734137 (28.08)	143726 (2.33)
Yes	56827 (0.92)	33613 (0.54)	21382 (0.35)	1832 (0.03)
COPD				
No	6115375 (99.03)	4232216 (68.54)	1738483 (28.15)	144676 (2.34)
Yes	59739 (0.97)	41821 (0.68)	17036 (0.28)	882 (0.01)
Hypertension				
No	5841788 (94.60)	4042477 (65.46)	1662482 (26.92)	136829 (2.22)
Yes	333326 (5.40)	231560 (3.75)	93037 (1.51)	8,729 (0.14)
Congestive heart failure				
No	6156440 (99.70)	4263386 (69.04)	1747958 (28.31)	145096 (2.35)
Yes	18674 (0.30)	10651 (0.17)	7,561 (0.12)	462 (0.01)
Diabetes				
No	6028038 (97.62)	4172160 (67.56)	1715613 (27.78)	140265 (2.27)
Yes	147076 (2.38)	101877 (1.65)	39906 (0.65)	5,293 (0.09)

Cox proportional hazards regression models were used to estimate the association between COVID-19 testing and time to mental health–related healthcare utilization for anxiety and depression, with results reported as hazard ratios (HRs) and 95% confidence intervals (CIs) for both the overall cohort and the propensity score–matched sample. Kaplan–Meier methods were used to estimate time-to-event functions, and differences between exposure groups were assessed using the log-rank test.

To address potential confounding by indication, propensity scores were calculated using age and sex and applied through matching with replacement, resulting in a matched sample of 664,233 individuals. This approach matched each exposed individual to the nearest comparator based on propensity score, allowing reuse of controls to improve balance. Covariates were selected *a priori* based on clinical relevance and prior literature, including sociodemographic characteristics, comorbidities, and measures of healthcare utilization, and were incorporated into both regression and propensity score models to enhance comparability between groups. All analyses were conducted using SAS Enterprise Guide 7.1 (SAS Institute, Cary, NC).

### Ethics approval

This study was approved by the University of Waterloo research ethics board (protocol reference no. 43910), and data use was authorized under section 45 of Ontario’s *Personal Health Information Protection Act*. ICES is a prescribed entity under section 45 of Ontario’s Personal Health Information Protection Act, which authorizes ICES to collect personal health information, without consent, for the purpose of health system evaluation and improvement.

## Results

The final study cohort was extracted as shown in Figure 1, while overall cohort characteristics at baseline are shown in Table 1. The demographic characteristics of propensity score matched sample are provided in the supplement (Supplementary Table S2).

Out of 6,175,114 individuals, there were 4,274,037 (69.21%) without a standard lab-based PCR test for COVID-19. Out of those who were tested for COVID-19 infection, there were 1,755,519 (28.43%) with negative results while 145,558 (2.36%) tested positive for COVID-19 during the study period. The mean age of the overall sample was 42.9 years with a standard deviation of 15.5. The number of females in the overall sample was higher (n = 3,189,737; 51.65%), as compared to males (n = 2,985,377; 48.35%). A higher proportion of individuals (n = 5,511,050; 89.25%) did not utilize health services for anxiety or depression-related issues as compared to those who contacted health services (n = 664,064; 10.75%) for these medical reasons. Out of those who contacted health services, the highest proportion of the individuals was 18–45 years of age (57.80%), followed by those between 46 and 65 years (32.62%) and 66–104 years (9.58%), respectively. In terms of sociodemographic status, the sample included 29.19% vs. 16.21% (Q1 vs. Q5) in dependency quintile, 24.61% vs. 17.29% (Q1 vs. Q5) in deprivation quintile, 22.56% vs. 22.63% (Q1 vs. Q5) in instability quintile, and 14.91% vs. 29.03% in ethnic diversity quintile (Table 1).

**FIGURE 2:**
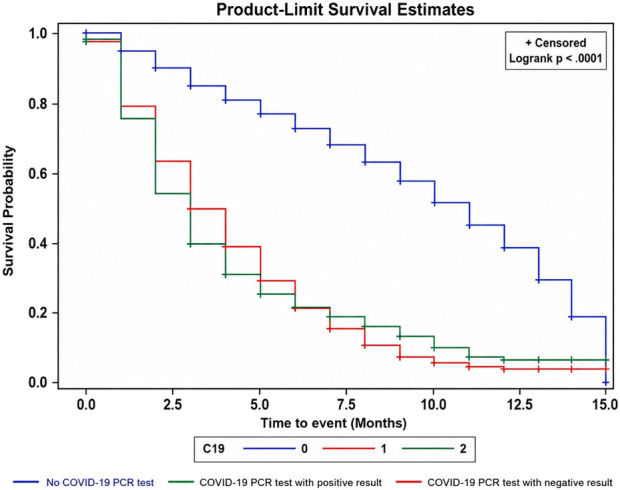
Kaplan-Meier plot of impact of COVID-19 PCR testing on time to anxiety- and/or depression-related service utilization (Ontario, Canada, 2021).

As seen in Figure 2, the time until the event (i.e., mental health service utilization), in months, was fastest in the group with negative COVID-19 test results, followed by the group with positive COVID-19 test results, both noticeably trailing those who never had standard lab-based PCR testing done for COVID-19 (Figure 2). For the negative group, the survival curve showed a gradual decrease in survival over time, whereas, for those without the COVID-19 PCR test, i.e., the control group, the curve followed the same pattern throughout. In the multivariable Cox proportional hazards model, COVID-19 testing, age, female sex, and chronic conditions were significantly associated with anxiety and depression related health service utilization (Table 2), with COVID-19 testing being the most important variable.

**TABLE 2:** Multivariable Cox regression analysis of factors associated with anxiety/depression related health services utilization among Ontario population for overall Sample (Ontario, Canada, 2021).

Variables	Crude HR (95% CI)	p-value	Adjusted HR (95% CI)	p-value
COVID-19 test status				
Covid positive	3.83 (3.76–3.91)	<0.0001	4.14 (4.39–4.44)	<0.0001
Covid negative	4.21 (4.18–4.24)	<0.0001	4.41 (4.06–4.22)	<0.0001
No PCR test	Ref	​	Ref	​
Age groups				
18–45 years	1.21 (1.20–1.22)	<0.0001	1.38 (1.36–1.39)	<0.0001
46–65 years	1.14 (1.13–1.15)	<0.0001	1.31 (1.30–1.33)	<0.0001
65–104 years	Ref	​	Ref	​
Sex				
Female	1.05 (1.05–1.06)	<0.0001	1.05 (1.04–1.05)	<0.0001
Male	Ref	Ref	Ref	Ref
Rural				
Yes	1.00 (0.99–1.00)	<0.0001	0.99 (0.98–1.00)	0.2682
No	Ref	​	Ref	​
Asthma				
Yes	1.07 (1.05–1.10)	<0.0001	1.00 (0.98–1.02)	0.7479
No	Ref	​	Ref	​
COPD				
Yes	0.94 (0.92–0.96)	<0.0001	0.91 (0.89–0.93)	<0.0001
No	Ref	​	Ref	​
Congestive heart failure				
Yes	0.69 (0.65–0.70)	<0.0001	0.43 (0.41–0.45)	<0.0001
No	Ref	​	Ref	​
Hypertension				
Yes	0.97 (0.96–0.98)	<0.0001	0.99 (0.98–1.00)	0.2763
No	Ref	​	Ref	​
Diabetes				
Yes	0.95 (0.94–0.97)	<0.0001	0.92 (0.91–0.94)	<0.0001
No	Ref	​	Ref	​
Ontario marginalization index Dependency quintile				
1	Ref	​	Ref	​
2	0.99 (0.98–1.00)	0.1920	1.00 (0.99–1.00)	0.9588
3	0.99 (0.98–1.00)	0.3700	1.00 (0.99–1.00)	0.7363
4	0.98 (0.98–0.99)	0.0023	0.99 (0.99–1.00)	0.7545
5	0.97 (0.97–0.98)	<0.0001	0.98 (0.97–0.99)	0.0002
Deprivation quintile				
1	Ref	​	Ref	​
2	0.99 (0.98–1.00)	0.0761	0.99 (0.99–1.00)	0.8050
3	0.99 (0.98–1.00)	0.7505	1.00 (0.99–1.01)	0.2907
4	0.98 (0.97–0.99)	0.0002	1.00 (0.99–1.00)	0.9674
5	0.97 (0.97–0.98)	<0.0001	1.00 (0.99–1.00)	0.9923
Instability quintile				
1	Ref	​	Ref	​
2	1.00 (0.99–1.00)	0.9996	0.99 (0.98–0.99)	0.0285
3	1.00 (0.99–1.00)	0.7777	0.99 (0.98–1.00)	0.0379
4	0.99 (0.99–1.00)	0.7811	0.97 (0.97–0.98)	<0.0001
5	0.98 (0.97–0.99)	<0.0001	0.96 (0.955–0.97)	<0.0001
Ethnic diversity quintile				
1	Ref	​	Ref	​
2	1.01 (1.00–1.02)	0.0060	1.00 (0.99–1.00)	0.9285
3	1.01 (1.00–1.01)	0.0151	0.99 (0.98–1.00)	0.1295
4	1.00 (0.99–1.01)	0.5133	0.98 (0.97–0.99)	0.0095
5	0.978 (0.97–0.98)	<0.0001	0.97 (0.96–0.98)	<0.0001

In the adjusted Cox proportional hazards model for the overall sample, the individuals who had PCR tests had a higher health services utilization for anxiety and depression during the pandemic, as compared to those who had no PCR testing done for COVID-19. The hazard ratios were aHR, 4.14; (95%) CI, 4.39–4.44 and aHR, 4.41; CI, 4.06–4.22; for COVID-19 test positive and COVID-19 test negative, respectively (Table 2). A higher health services utilization for anxiety and depression was observed among females (aHR, 1.05; CI, 1.04–1.05), individuals in the 18–45 years age group (aHR 1.38; CI, 1.36–1.39) as well as individuals in the 46–65 years age group (aHR, 1.31; CI, 1.30–1.33).

In the Cox proportional hazards model in the propensity score matched sample, the individuals who tested positive for COVID-19 (aHR, 6.37; CI, 6.25–6.50) had the higher health services utilization for anxiety and depression during the pandemic, followed by those who tested negative for COVID-19 (aHR, 5.91; CI, 5.87–5.95) (Table 3). Also, a higher health services utilization was noted among young individuals, females, and those residing in rural areas. Moreover, higher services utilization was observed for socioeconomic status, with material deprivation quintile significantly associated with anxiety and depression. For the neighborhood-level income quintile, similar risks of anxiety and depression were found among those in Q3-Q5 of the material deprivation quintile (Table 3).

**TABLE 3:** Multivariable Cox regression analysis of factors associated with anxiety/depression related health services utilization among Ontario population after propensity scores computation (Ontario, Canada, 2021).

Variables	Crude HR (95% CI)	p-value	Adjusted HR (95% CI)	p-value
COVID-19 test status				
Covid positive	6.34 (6.21–6.46)	<0.0001	6.37 (6.25–6.50)	<0.0001
Covid negative	5.88 (5.84–5.92)	<0.0001	5.91 (5.87–5.95)	<0.0001
No PCR test	Ref	​	Ref	​
Age groups				
18–45 years	1.04 (1.03–1.05)	<0.0001	1.000 (0.977–0.996)	<0.0001
46–65 years	1.00 (0.991–1.01)	0.7066	0.980 (0.971–0.989)	<0.0001
65–104 years	Ref	​	Ref	​
Sex				
Female	1.00 (0.99–1.00)	0.0080	1.039 (1.034–1.044)	<0.0001
Male	Ref	Ref	Ref	Ref
Rural				
Yes	1.02 (1.02–1.03)	<0.0001	1.02 (1.01–1.03)	<0.0001
No	Ref	​	Ref	​
Asthma				
Yes	1.04 (1.02–1.06)	<0.0001	0.94 (0.92–0.96)	<0.0001
No	Ref	​	Ref	​
COPD				
Yes	1.00 (0.98–1.03)	0.3877	0.99 (0.97–1.01)	0.4776
No	Ref	​	Ref	​
Congestive heart failure				
Yes	1.21 (1.16–1.27)	<0.0001	1.09 (1.05–1.14)	<0.0001
No	Ref	​	Ref	​
Hypertension				
Yes	1.01 (1.00–1.02)	0.0014	1.00 (0.99–1.01)	0.2208
No	Ref	​	Ref	​
Diabetes				
Yes	1.00 (0.98–1.01)	0.6820	0.99 (0.97–1.00)	0.4915
No	Ref	​	Ref	​
Ontario marginalization index				
Dependency quintile				
1	Ref	​	Ref	​
2	0.99 (0.99–1.00)	0.6058	0.99 (0.99–1.00)	0.3154
3	1.00 (0.99–1.00)	0.7809	0.99 (0.99–1.00)	0.5263
4	0.99 (0.99–1.00)	0.6277	1.00 (0.99–1.00)	0.8722
5	1.00 (0.99–1.00)	0.9174	0.99 (0.99–1.00)	0.8452
Deprivation quintile				
1	Ref	​	Ref	​
2	0.99 (0.98–1.00)	0.3369	1.00 (0.99–1.01)	0.1062
3	1.00 (0.99–1.00)	0.6515	1.01 (1.00–1.02)	<0.0001
4	0.99 (0.98–1.00)	0.0557	1.02 (1.01–1.03)	<0.0001
5	0.98 (0.97–0.99)	<0.0001	1.03 (1.02–1.04)	<0.0001
Instability quintile				
1	Ref	​	Ref	​
2	1.00 (0.99–1.01)	0.5453	0.98 (0.98–0.99)	0.0044
3	1.00 (0.99–1.01)	0.4752	0.98 (0.97–0.99)	0.0001
4	1.00 (0.99–1.00)	0.7743	0.97 (0.96–0.97)	<0.0001
5	0.98 (0.97–0.99)	<0.0001	0.95 (0.94–0.95)	<0.0001
Ethnic diversity quintile				
1	Ref	​	Ref	​
2	1.00 (0.99–1.00)	0.9959	0.99 (0.98–1.00)	0.1657
3	0.99 (0.98–0.99)	0.0127	0.97 (0.96–0.98)	<0.0001
4	0.98 (0.97–0.98)	<0.0001	0.97 (0.96–0.98)	<0.0001
5	0.96 (0.96–0.97)	<0.0001	0.97 (0.96–0.98)	<0.0001

In the Cox proportional hazards model stratified by age groups, the health services utilization for anxiety and depression was highest among younger adults (aHR, 5.56; CI, 5.51–5.61), as compared to the middle-age group (aHR, 4.05; CI, 4.00–4.09) and older adults (aHR, 1.59; CI, 1.55–1.62), who tested negative with COVID-19 (Supplementary Table S1). Females were at higher risk as compared to males across all age groups (Table 3). Rural residence was only statistically significant among younger adults (aHR, 1.02; CI, 1.01–1.03) as compared to other age groups, though the effect is small. Among chronic conditions, asthma and hypertension were observed to be associated with anxiety and depression-related visits and hospitalization, across young and middle age groups (Supplementary Table S1).

## Discussion

### Main findings

This study examined differences in first recorded mental health service utilization for anxiety and depression between individuals who did and did not undergo COVID-19 PCR testing in a large population-based cohort during the pandemic. We observed higher utilization among individuals who underwent testing, reflecting underlying differences between tested and untested populations rather than a direct effect of testing itself, with adjusted hazard ratios of 6.37 (95% CI 6.25–6.50) for PCR-positive individuals and 5.91 (95% CI 5.87–5.95) for PCR-negative individuals. Higher utilization was particularly evident among younger individuals, females, and those residing in rural areas, with additional socioeconomic gradients indicating greater use among more deprived populations. The magnitude and similarity of effect estimates across PCR-positive and PCR-negative groups suggest that testing functions primarily as a proxy for underlying vulnerability and healthcare-seeking behavior rather than a causal determinant of mental health outcomes.

### Comparison with existing literature

The observed patterns of mental health–related healthcare utilization may be consistent with broader reports from the pandemic; however, these findings cannot distinguish between true increases in mental health burden and differences in healthcare-seeking behavior. The increased mental health–related healthcare utilization observed in this study is consistent with evidence on the psychological impact of the COVID-19 pandemic, driven by factors such as uncertainty, social isolation, and economic disruption [21, 22]. Higher risks among younger individuals and females align with prior studies reporting disproportionate mental health impacts in these groups [28–32].

Social determinants of health play a key role in shaping these outcomes. Individuals in lower-income groups experienced greater mental health challenges, consistent with evidence linking financial stress, job loss, and poverty to anxiety and depression [33–35]. Similarly, we observed higher risks among rural residents, which may reflect barriers to care, social isolation, and limited access to mental health services [34, 36].

Chronic conditions, including asthma, hypertension, and congestive heart failure, were also associated with increased mental health service utilization. These conditions, identified as risk factors for severe COVID-19 illness, may contribute to heightened psychological distress, further exacerbated by disruptions in routine care during the pandemic [37, 38].

Overall, these findings highlight the disproportionate burden of mental health service utilization among socioeconomically and clinically vulnerable populations during the pandemic [37, 39].

### What does this study add to existing knowledge?

This study characterizes patterns of mental health–related healthcare utilization among individuals who engaged with COVID-19 testing during the pandemic rather than establishing a causal relationship between testing and mental health outcomes. The findings indicate higher utilization among tested individuals; however, the magnitude of these associations is unusually large for observational mental health research and is unlikely to represent causal effects. Instead, these patterns are most plausibly explained by substantial residual confounding, including underlying differences in healthcare-seeking behavior and psychosocial vulnerability between individuals who seek testing and those who do not. The similarity of effect estimates across PCR-positive and PCR-negative groups further supports the interpretation that testing functions primarily as a proxy for underlying vulnerability rather than a direct determinant of mental health outcomes. Accordingly, these results should be interpreted as descriptive of healthcare utilization patterns during the pandemic rather than evidence of a direct psychological impact of COVID-19 testing.

Individuals undergoing testing may differ systematically in ways that predispose them to higher mental health service use, including greater baseline health anxiety, increased psychological vulnerability, and a higher propensity to engage with healthcare services [40]. These factors may contribute to residual confounding, even after adjustment for measured variables. Surveillance bias is also a likely contributor, as individuals interacting with the healthcare system through testing may be more likely to have mental health conditions identified and recorded. The similar effect estimates observed in both PCR-positive and PCR-negative groups further support the interpretation that testing itself is not the primary driver, but rather a marker of shared underlying characteristics among those who access testing [41]. Reverse causality must also be considered, as individuals with pre-existing anxiety or heightened concern about COVID-19 may have been more likely to seek testing.

Taken together, these findings suggest that COVID-19 testing serves as an indicator of a population with elevated underlying risk of mental health service utilization, rather than a direct causal determinant of mental health outcomes.

### What are the key implications for public health practice or policy?

The observed disparities in mental health–related healthcare utilization among socioeconomically disadvantaged populations point to important inequities in patterns of healthcare engagement during the pandemic. However, these findings should not be interpreted as evidence of greater underlying mental health need, as they may reflect differences in healthcare access, utilization, and help-seeking behavior rather than true differences in burden. Instead, the results are best understood as identifying populations with higher levels of interaction with the healthcare system during this period.

While causal inferences cannot be drawn, these patterns may still be useful for public health planning by highlighting groups that were more frequently represented in mental health service use during the pandemic. Monitoring trends in healthcare utilization can support resource planning and help ensure that services are responsive to populations engaging with care. However, caution is required in translating these findings into policy, as observed differences may not correspond to underlying need.

Future research should prioritize disentangling the roles of psychosocial factors, healthcare-seeking behavior, and structural inequalities in shaping observed patterns of mental health service utilization [42]. The findings highlight the importance of interpreting administrative healthcare data within the broader context of social and behavioral responses to the pandemic. Strengthening mental health systems and incorporating mental health considerations into pandemic preparedness remain important goals, but should be informed by evidence that distinguishes true need from patterns of healthcare engagement.

### Strengths and limitations

To our knowledge, this is one of the largest population-based studies examining the association between COVID-19 testing and mental health–related healthcare utilization in Ontario. The use of linked administrative health data within a universal healthcare system allowed for comprehensive capture of physician visits, emergency department encounters, and psychiatric hospitalizations, enhancing the generalizability of the findings. Additionally, the application of propensity score matching improved comparability between tested and untested individuals by balancing measured sociodemographic and clinical characteristics.

However, several important limitations should be considered when interpreting these findings. The outcome reflects the first recorded use of mental health–related services during the study period rather than incident diagnoses of anxiety or depression. Accordingly, the results represent patterns of healthcare utilization, which may include both new-onset and pre-existing but previously unrecorded conditions, rather than true incidence of mental illness.

Moreover, despite the use of propensity score matching, residual confounding remains likely, particularly due to unmeasured psychosocial factors. Variables such as employment status, income loss, social isolation, media exposure, baseline anxiety traits, and individual propensity for healthcare-seeking were not available in administrative data. These factors are strongly associated with both the likelihood of undergoing COVID-19 testing and the use of mental health services. Individuals experiencing greater psychological distress or socioeconomic disruption during the pandemic may have been both more likely to seek testing and more likely to access care. As a result, the observed associations may be inflated, reflecting underlying vulnerability rather than the effect of testing itself.

The similar magnitude of associations in both PCR-positive and PCR-negative groups further supports this interpretation, suggesting that testing is unlikely to be a direct causal driver of increased mental health service utilization. Instead, testing may act as a marker of heightened vulnerability, capturing individuals with greater health anxiety, perceived risk, or engagement with the healthcare system. Effect sizes of this magnitude are unlikely to be explained by measured confounders alone and likely reflect selection processes and unmeasured differences between tested and untested populations.

The administrative data captures only individuals who interact with the healthcare system, potentially excluding those with untreated or unrecognized mental health conditions. This may introduce detection (surveillance) bias, as individuals who seek testing are also more likely to have mental health concerns identified and recorded.

Additional limitations include the exclusion of individuals with missing demographic data, limited information on certain populations (e.g., immigrants and Indigenous communities), and the inability to assess severity or chronicity of mental health conditions. Finally, the study period did not extend into later phases of the pandemic, which may limit the assessment of longer-term mental health impacts.

### Conclusion

Our study identifies higher mental health–related healthcare utilization among individuals who underwent COVID-19 PCR testing for anxiety and depression. These patterns reflect differences between tested and untested populations and should not be interpreted as evidence of a direct psychological impact of testing. The similar magnitude of associations observed in PCR-positive and PCR-negative individuals strongly suggests that testing functions primarily as a proxy for underlying vulnerability and healthcare-seeking behavior rather than a causal determinant of mental health outcomes. These findings pertain to patterns of healthcare utilization rather than new-onset mental illness. As such, the results are best understood as descriptive of healthcare engagement during the pandemic, highlighting the importance of interpreting administrative data within the context of underlying differences in access, utilization, and psychosocial factors. Incorporating mental health considerations into pandemic preparedness remains important but should be guided by evidence that distinguishes true need from patterns of healthcare use.

## Data Availability

The data set from this study is held securely in coded form at ICES. While data sharing agreements prohibit ICES from making the data set publicly available, access may be granted to those who meet pre-specified criteria for confidential access, available at https://www.ices.on.ca/DAS. The full data set creation plan and underlying analytic code are available from the authors upon request, understanding that the computer programs may rely upon coding templates or macros that are unique to ICES and are therefore either inaccessible or may require modification.
