# Supplementary material for: COVID-19 testing and mental health service utilization in Ontario: a population-based cohort study
Source: Int J Public Health. 2026 Jun 2;71:1609134. doi: 10.3389/ijph.2026.1609134 (PMC13220775; doi:10.3389/ijph.2026.1609134)
Supplement: Supplementary file 1 [file Supplementaryfile1.docx]

**Table S1: Multivariable Cox regression analysis of factors associated with anxiety/depression related health services utilization among Ontario population stratified by age groups.**

| **Variables** | **Young Adults**  (18-45 yrs.) | **Middle-aged adults**  (46-65 yrs.) | **Older adults**  (65-104 yrs.) |
| --- | --- | --- | --- |
| COVID-19 Test Status |  |  |  |
| No PCR | Ref | Ref | Ref |
| Covid Negative | 5.56 (5.51-5.61)** | 4.05 (4.00- 4.09)** | 1.59 (1.55-1.62)** |
| Covid Positive | 5.46 (5.33-5.60)** | 4.24 (4.10- 4.38)** | 0.95 (0.88-1.03) |
| Sex |  |  |  |
| Female | 1.05 (1.05- 1.06)** | 1.05 (1.04-1.06)** | 1.10 (1.08-1.12)** |
| Male | Ref | Ref | Ref |
| Rural |  |  |  |
| Yes | 1.02 (1.01-1.03)** | 0.99 (0.97-1.00) | 0.94 (0.91-0.97) |
| No | Ref | Ref | Ref |
| Asthma |  |  |  |
| Yes | 1.06 (1.04-1.09)** | 1.08 (1.04-1.12)** | 1.09 (1.01-1.17)* |
| No | Ref | Ref | Ref |
| COPD |  |  |  |
| Yes | 0.99 (0.95-1.03) | 0.99 (0.96- 1.02) | 0.92 (0.88-0.97) |
| No | Ref | Ref | Ref |
| Congestive Heart Failure |  |  |  |
| Yes | 1.00 (0.87-1.14)* | 0.88 (0.83-0.94) | 0.60 (0.56-0.64) |
| No | Ref | Ref | Ref |
| Hypertension |  |  |  |
| Yes | 1.02 (1.00-1.04)* | 1.02 (1.00-1.03)* | 1.00 (0.97-1.02)* |
| No | Ref | Ref | Ref |
| Diabetes |  |  |  |
| Yes | 1.01 (0.98-1.03)* | 0.97 (0.95-1.00) | 0.87 (0.84-0.91) |
| No | Ref | Ref | Ref |
| Ontario Marginalization Index  Dependency quintile |  |  |  |
| 1 | Ref | Ref | Ref |
| 2 | 1.00 (0.99-1.01) | 0.99 (0.98-1.00) | 0.98 (0.95-1.01) |
| 3 | 1.00 (0.99-1.01) | 0.99 (0.98-1.01) | 0.99 (0.97-1.02) |
| 4 | 1.00 (0.99-1.01) | 0.99 (0.97-1.00) | 0.98 (0.96-1.01) |
| 5 | 1.01 (1.00-1.02) | 0.98 (0.97-0.99) | 0.96 (0.94-0.99) |
| Deprivation quintile |  |  |  |
| 1 | Ref | Ref | Ref |
| 2 | 0.98 (0.97-0.99) | 1.00 (0.98-1.01) | 0.99 (0.96-1.01) |
| 3 | 0.99 (0.98-1.00) | 0.99 (0.98-1.00) | 0.97 (0.95-1.00) |
| 4 | 0.98 (0.97-0.99) | 0.98 (0.97-0.99) | 0.94 (0.91-0.96) |
| 5 | 0.98 (0.97-0.99) | 0.96 (0.95-0.97) | 0.93 (0.91-0.96) |
| Instability quintile |  |  |  |
| 1 | Ref | Ref | Ref |
| 2 | 1.00 (0.99-1.01) | 0.99 (0.98-1.00) | 0.99 (0.96-1.02) |
| 3 | 1.01 (1.00-1.02) | 0.99 (0.97-1.00) | 0.98 (0.95-1.01) |
| 4 | 1.01 (1.00-1.02) | 0.98 (0.97-0.99) | 0.96 (0.93-0.98) |
| 5 | 1.00 (0.99-1.01) | 0.96 (0.94-0.97) | 0.95 (0.93-0.98) |
| Ethnic diversity quintile |  |  |  |
| 1 | Ref | Ref | Ref |
| 2 | 1.00 (0.99-1.01) | 1.01 (1.00-1.03) | 1.02 (0.99-1.05) |
| 3 | 1.00 (0.99-1.01) | 1.00 (0.98-1.01) | 1.02 (1.00-1.05) |
| 4 | 0.98 (0.97-0.99) | 0.99 (0.98-1.01) | 1.01 (0.98-1.04) |
| 5 | 0.95 (0.94-0.96) | 0.98 (0.96-0.99) | 0.98 (0.95-1.01) |

****** p-value < .0001 ***** p-value < .01

**Table S2**: **Demographic characteristics and morbidity information of the study population cohort in Ontario (Jan 2020- Mar 2021) after propensity score matching**

| **Demographics & Morbidity information** | **Total sample** | **Individuals without PCR COVID-19 tests** | | **PCR test with negative COVID-19 result** | **PCR test with positive COVID-19**  **result** |
| --- | --- | --- | --- | --- | --- |
| Total n (%) | 664233 (100) | 485780 (73.13) | | 167616 (25.23) | 10837 (1.63) |
| Mean Age Yrs. (±SD) | 42.82 (14.45) | 43.43 (14.51) | | 41.09 (14.13) | 41.96 (14.21) |
| Anxiety/depression related health service utilization | | |  |  |  |
| No | 169 (0.03) | 127 (0.02) | | 35 (0.01) | 7 (0.00) |
| Yes | 664064 (99.97) | 485653 (73.11) | | 167581 (25.23) | 10837 (1.63) |
| Age groups n (%) | | |  |  |  |
| 18-45 yrs. | 393466 (59.24) | 278653 (41.95) | | 108199 (16.29) | 6614 (1.00) |
| 46-65 yrs. | 217627 (32.76) | 165319 (24.89) | | 48715 (7.33) | 3593 (0.54) |
| 66-104 yrs. | 53140 (8.00) | 41808 (6.29) | | 10702 (1.61) | 630 (0.09) |
| Sex n (%) | | |  |  |  |
| Female | 419725 (63.19) | 300302 (45.21) | | 112270 (16.90) | 7153 (1.08) |
| Male | 244508 (36.81) | 185478 (27.92) | | 55346 (8.33) | 3684 (0.55) |
| Rural n (%) | | |  |  |  |
| No | 608616 (91.63) | 443770 (66.81) | | 154394 (23.24) | 10452 (1.57) |
| Yes | 55617 (8.37) | 42010 (6.32) | | 13222 (1.99) | 385 (0.06) |
| Ontario Marginalization Index (%) | | |  |  |  |
| Dependency quintile |  |  | |  |  |
| 1 | 18723 (28.19) | 135382 (20.38) | | 48160 (7.25) | 3690 (0.56) |
| 2 | 138335 (20.83) | 100526 (15.13) | | 35385 (5.33) | 2424 (0.36) |
| 3 | 118132 (17.78) | 86546 (13.03) | | 29713 (4.47) | 1873 (0.28) |
| 4 | 108911 (16.40) | 80507 (12.12) | | 26882 (4.05) | 1522 (0.23) |
| 5 | 111623 (16.80) | 82819 (12.47) | | 27476 (4.14) | 1328 (0.20) |
| Deprivation quintile | | |  |  |  |
| 1 | 161494 (24.31) | 116409 (17.53) | | 42968 (6.47) | 2117 (0.32) |
| 2 | 139878 (21.06) | 102343 (15.41) | | 35549 (5.35) | 1986 (0.30) |
| 3 | 123657 (18.62) | 90467 (13.62) | | 31000 (4.67) | 2190 (0.33) |
| 4 | 116977 (17.61) | 85995 (12.95) | | 28899 (4.35) | 2083 (0.31) |
| 5 | 122227 (18.40) | 90566 (13.63) | | 29200 (4.40) | 2461 (0.37) |
| Instability quintile | | |  |  |  |
| 1 | 135253 (20.36) | 99928 (15.04) | | 32483 (4.89) | 2842 (0.43) |
| 2 | 120248 (18.10) | 88675 (13.35) | | 29799 (4.49) | 1774 (0.27) |
| 3 | 118297 (17.81) | 87188 (13.13) | | 29478 (4.44) | 1631 (0.25) |
| 4 | 123344 (18.57) | 89990 (13.55) | | 31491 (4.74) | 1863 (0.28) |
| 5 | 167091 (25.16) | 119999 (18.07) | | 44365 (6.68) | 2727 (0.41) |
| Ethnicity diversity quintile | | |  |  |  |
| 1 | 97384 (14.66) | 72816 (10.96) | | 23872 (3.59) | 696 (0.10) |
| 2 | 113164 (17.04) | 82827 (12.47) | | 29246 (4.40) | 1091 (0.16) |
| 3 | 132414 (19.93) | 95473 (14.37) | | 35266 (5.31) | 1675 (0.25) |
| 4 | 154578 (23.27) | 111325 (16.76) | | 40691 (6.13) | 2562 (0.39) |
| 5 | 166693 (25.10) | 123339 (18.57) | | 38541 (5.80) | 4813 (0.72) |
| Asthma | | |  |  |  |
| No | 654826 (98.58) | 497734 (72.22) | | 164498 (24.77) | 10594 (1.59) |
| Yes | 9407 (1.42) | 6046 (0.91) | | 3118 (0.47) | 243 (0.04) |
| COPD | | |  |  |  |
| No | 655047 (98.62) | 479059 (72.12) | | 165263 (24.88) | 10725 (1.61) |
| Yes | 9186 (1.38) | 6721 (1.01) | | 2353 (0.35)1 | 112 (0.02) |
| Hypertension | | |  |  |  |
| No | 623147 (93.81) | 455802 (68.62) | | 157383 (23.69) | 9962 (1.50) |
| Yes | 41086 (6.19) | 29978 (4.51) | | 10233 (1.54) | 875 (0.13) |
| Congestive heart failure | | |  |  |  |
| No | 662106 (99.68) | 484455 (72.93) | | 166869 (25.12) | 10782 (1.62) |
| Yes | 2127 (0.32) | 1325 (0.20) | | 747 (0.11) | 55 (0.01) |
| Diabetes |  |  | |  |  |
| No | 647373 (97.46) | 473590 (71.30) | | 163410 (24.60) | 10373 (1.56) |
| Yes | 16860 (2.54) | 12190 (1.84) | | 4206 (0.63) | 464 (0.07) |

**ADDITIONAL SUPPLEMENTARY INFORMATION**

**Table S3: Details of ICES data dictionaries utilized in the study.**

|  | **Data Dictionaries (Jan 2020-Mar 2021)** | | **Category** |
| --- | --- | --- | --- |
| 1. | C19INTGR | COVID19 Integrated Testing Data | Health Services |
| 2. | RPDB | Registered Persons Database | Population& Demographic |
| 3. | Asthma | Ontario Asthma Dataset | ICES-Derived Cohort |
| 4. | COPD | Chronic Obstructive Pulmonary Disease | ICES-Derived Cohort |
| 5. | HYPER | Ontario Hypertension dataset | ICES-Derived Cohort |
| 6. | ODD | Ontario Diabetes Dataset | ICES-Derived Cohort |
| 7. | CHF | Congestive Heart Failure | ICES-Derived Cohort |
| 8. | OMHRS | Ontario Mental Health Reporting System | Health Services |
| 9. | DAD | Discharge Abstract Database DAD | Health Services |
| 10. | OHIP | Ontario Health Insurance Plan Claims Database | Health Services |
| 11. | NACRS | National Ambulatory Care Reporting System | Health Services |
| 12. | ORGD | Vital Statistics-Death | Population& Demographic |
| 11. | ONMARG | Ontario Marginalization Index | Coding & Geography |

**Table S4.** Description of cohort definition and variables within the study cohort (administrative database), and the codes used.

| **Variable name** | | **Source of data** | **Description** | **Variable name in database** | **Database codes** |
| --- | --- | --- | --- | --- | --- |
| **exposure** | | | | | |
| Covid result based on PCR test | | C19INTGR | COVID19 Integrated Testing Data  Covid standard lab-based PCR test Result between January 2020 and March 31, 2021. | COVIDRESULT | P= Positive  N=Negative |
| **Outcome/Event** | | | | | |
| Health services utilization for a mental health reason | | DAD  OMHRS  NACRS  OHIP | - Primary diagnosis of either anxiety or depression from a psychiatric bed (OMHRS) or medical bed (DAD) between January 1, 2020, and March 31, 2021, * OR - At least one emergency department (NACRS) or physician visits (OHIP) during study period for Anxiety/Depression from January 1, 2020, and March 31, 2021* | DX10CODE1 (DAD) MHA_DXGROUP (OMHRS) DX10CODE (NACRS)  DXCODE (OHIP) | ICD-10 Codes (DAD) and  (NACRS) to identify Anxiety /Depression  DSM-V criteria (OMHRS)  OHIP diagnostic codes |
| **All-cause Mortality** | | | | | |
| Vital Statistics-Death | | ORGD | All cause mortality between January 1, 2020, and March 31, 2021 | DTHDATE | 1= died  0=Alive |
| **Demographic Factors** | | | | | |
| Age at index date | | RPDB | Age at index date | BDATE | Variable calculated based on index date and birth date |
| Sex | | RPDB | Recorded sex | SEX | 1 = Male  2 = Female |
| Rural residence | | RPDB | Rural place of residence, defined as residing in a community of ≤ 10,000 | RURAL | 1 = Rural flag  0 = non-Rural |
| **Ontario Marginalization Index** | | | | | |
| Residential Instability | | ONMARG | Neighbourhood-level indicator of residential instability constructed based on census data | INSTABILITY_Q | 1 = Least Marginalized 5 = Most Marginalized |
| Material Deprivation | | ONMARG | Neighbourhood-level indicator of material deprivation constructed based on census data | DEPRIVATION_Q | 1 = Least Marginalized 5 = Most Marginalized |
| Ethnic Diversity | | ONMARG | Neighbourhood-level indicator of ethnic concentration constructed based on census data | ETHNICDIV_Q | 1 = Least Marginalized 5 = Most Marginalized |
| Dependency | | ONMARG | Neighbourhood-level indicator of social dependency constructed based on census data | DEPENDENCY_Q | 1 = Least Marginalized 5 = Most Marginalized |
| **Chronic Diseases** | | | | | |
| Asthma | | ASHTHMA | Ontario Asthma dataset | ASTHMA | 1= Diagnosed with Asthma  0 = No asthma |
| CHF | | CHF | Congestive Heart Failure | CHF | 1= Diagnosed with CHF  0 = No CHF |
| HTN | | HTN | Hypertension | Hyper | 1= Diagnosed with HTN  0 = No HTN |
| COPD | | COPDC | Chronic Obstructive Pulmonary Disease | COPD | 1= Diagnosed with COPD  0 = No COPD |
|  | |  |  |  |  |
| Diabetes | | ODD | Ontario Diabetes Dataset | ODD | 1= Diagnosed with Diabetes  0 = No Diabetes |
| **DAD Variables** | | | | | |
| Diagnosis of medical condition | | DAD | To identify the individuals with chronic conditions of interest | DX10CODE1 (DAD) | All ICD-10 Codes for Anxiety and Depression |
| Hospital Admission Date | | DAD | Date of Hospital Admission | DAD_ADMNDATE |  |
| **NACRS Variables** | | | | | |
| Registration Date | |  | NACRS Registration Date | REGDATE |  |
| Diagnosis of medical condition | | NACRS | To identify the individuals with chronic conditions of interest | DX10CODE1 (NACRS) | All ICD-10 Codes for chronic conditions of interest |
| **OMHRS Variables** | | | | | |
| Diagnosis of Mental Illnesses | OMHRS | | General Mental Health and Addiction Hospitalization Episodes | MHA_DXGROUP | ANX = Anxiety MDIS_DEP = Mood Disorders - Depressive Based on DSM-V criteria |
| Hospital Admission due to mental reason | OMHRS /DAD | | Date of Episode Admission | EPI_ADNDATE |  |
| **OHIP Variables** | | | | | |
| Diagnosis of medical condition | OHIP | | OHIP Diagnosis Code | DXCODE | 300 = Anxiety neurosis, hysteria, neurasthenia, obsessive compulsive neurosis, reactive depression  311 = Depressive or other non-psychotic disorders, not elsewhere classified |
| Service date |  | | Date on which service was provided | SERVDATE |  |

**Table S5. List of definitions of comorbidities**

| **Comorbidity** | **Case definition** | **Codes** | **Validation** |
| --- | --- | --- | --- |
| Asthma^^[[1]](#footnote-1)^^ | At least one hospitalization admission with a diagnosis of asthma or two physician claims of asthma diagnosis in a two-year period preceding the index date | ICD-9/OHIPDX: 493  ICD-10: J45, J46 | Sensitivity = 81%  Specificity = 81% |
| COPD^^[[2]](#footnote-2)^^ | At least 1 physician billings within 2 years or one hospitalization with a COPD diagnosis code prior to index date. | ICD-9/OHIPDX: 491, 492, 496  ICD-10: J41, J42, J43, J44 | Sensitivity = 85%  Specificity = 79% |
| Hypertension^^[[3]](#footnote-3)^^ | At least one hospital admission with a diagnosis of hypertension or two physician claims for hypertension within two years prior to index date. Cases of gestational hypertension would be excluded. | ICD-9/OHIPDX: 401x, 402x, 403x, 404x, 405x  ICD-10: I10, I11, I12, I13, I15 | Sensitivity = 73%  Specificity = 95% |
| Diabetes^^[[4]](#footnote-4)^^ | Age = 18:  At least four physician claims with a diabetes diagnosis code in a two-year period prior to index date OR at least one physician claims with a diabetes fee code.  Age 19+:  At least one hospital admission with a diagnosis of diabetes or two physician claims for diabetes or one prescription claim for diabetes medications within 1 year. Cases of gestational diabetes would be excluded. | ICD-9/OHIPDX: 250  ICD-10: E10, E11, E13, E14  OHIP Feecode: Q040, K029, K0303, K045, K046  ODB DINS: INSULIN or ORAL ANTI-GLYCEMICS | Age =18:  Sensitivity: 83%  Specificity: 99%  Age 19+  Sensitivity = 90% Specificity = 98% |
| Congestive heart failure^^[[5]](#footnote-5)^^ | At least one hospital admission with a congestive heart failure diagnosis, one physician claim/emergency department visit with a congestive heart failure diagnosis followed within one year by a second record from either source or one hospital admission. | ICD-9/OHIPDX: 428  ICD-10: I500, I501, I509 | Sensitivity = 85%  Specificity = 97% |
| Anxiety & Depression | Primary diagnosis of either anxiety or depression from a psychiatric bed (OMHRS) or medical bed (DAD) between January 1, 2020, and March 31, 2021, * OR  At least one emergency department (NACRS) or physician visits (OHIP) during study period for Anxiety/Depression from January 1, 2020, and March 31, 2021* | Anxiety disorders  DSM-V  ICD-9: 300.0, 300.2, 300.3, 309.8,308.3  ICD-10: F41.0, F41.1,F41.2. F41.3, F41.8, F41.9  Depression  DSM-V  ICD-9: 296.2, 296.3, 300.4, 311  ICD-10: F32.0, F32.1,F32.1, F32.2, , F32.3, F32.8, F32.9  F33.0, F33.1, F33.2, F33.3, F33.8,F33.9 | Not validated |

1. Gershon AS, Wang C, Guan J, Vasilevska-Ristovska J, Cicutto L, To T. Identifying patients with physician-diagnosed asthma in health administrative databases. Canadian respiratory journal 2009; 16(6): 183-8. [↑](#footnote-ref-1)
2. Gershon A, Wang C, Guan J, Vasilevska-Ristovska J, Cicutto L, To T. Identifying individuals with physician diagnosed COPD in health administrative databases. *COPD: Journal of Chronic Obstructive Pulmonary Disease* 2009; **6**(5): 388-94 [↑](#footnote-ref-2)
3. Tu K, Campbell NR, Chen Z-L, Cauch-Dudek KJ, McAlister FA. Accuracy of administrative databases in identifying patients with hypertension. *Open medicine* 2007; **1**(1): e18 [↑](#footnote-ref-3)
4. Lipscombe LL, Hwee J, Webster L, Shah BR, Booth GL, Tu K. Identifying diabetes cases from administrative data: a population-based validation study. *BMC health services research* 2018; 18(1): 1-8. [↑](#footnote-ref-4)
5. Schultz SE, Rothwell DM, Chen Z, Tu K. Identifying cases of congestive heart failure from administrative data: a validation study using primary care patient records. *Chronic diseases and injuries in Canada* 2013; 33(3). [↑](#footnote-ref-5)
